# Differential stress responses of immunoisolated human islets embedded in pancreatic extracellular matrix under static and free-fall dynamic conditions

**DOI:** 10.1177/20417314251383295

**Published:** 2025-10-27

**Authors:** Isaura Borges-Silva, Marluce da Cunha Mantovani, Minh Danh Anh Luu, Alan Gorter, Theo Borghuis, Naschla Gasaly, Mari Cleide Sogayar, Paul deVos, Marina Trombetta-Lima

**Affiliations:** 1 Department of Pharmaceutical Technology and Biopharmacy, Groningen Research Institute of Pharmacy, University of Groningen, Groningen, The Netherlands; 2 Department of Pathology and Medical Biology, University of Groningen, and University Medical Center Groningen, Groningen, The Netherlands; 3 Cell and Molecular Therapy NUCEL Group, School of Medicine, University of São Paulo, Brazil; 4 Division for Support of Training, Research and Innovation (DTAPEPI), School of Medicine, University of São Paulo, Brazil; 5 Department of Biochemistry, Chemistry Institute, University of São Paulo, Brazil; 6 Faculty of Science and Engineering, Maastricht Universit, The Netherlands

**Keywords:** dynamic culture, free-fall culture, rotating wall vessel device, ER-stress, human islets, extracellular matrix

## Abstract

Pancreatic islet transplantation offers great promise for the treatment of type 1 diabetes, yet the functional decline of islets after isolation remains a major obstacle. Increasing evidence highlights the endoplasmic reticulum (ER) as a critical regulator of islet cell survival under stress. We explored how ex vivo culture conditions affect encapsulated islet resilience under ER-stress. Two conditions were assessed: (i) incorporation of decellularized porcine pancreatic extracellular matrix (ECM) into alginate microcapsules, and (ii) free-fall dynamic pre-conditioning culture. Human islets were encapsulated in alginate with or without ECM, cultured under static or dynamic conditions, and exposed to acute ER-stress followed or not by a recovery period. Dynamic culture preserved viability and enhanced glucose responsiveness. ECM-containing capsules showed reduced inflammatory marker expression, while encapsulation in alginate-only capsules led to more pronounced changes associated with ECM remodeling. Under ER-stress, the dynamic culture, especially combined with ECM, maintained cell function and reduced cell death. Gene profiles indicated improved stress adaptation and ECM remodeling. These results highlight ECM enrichment and dynamic culture as good strategies to maintain islet survival and functionality.

## Graphical abstract

**Graphical Abstract:**
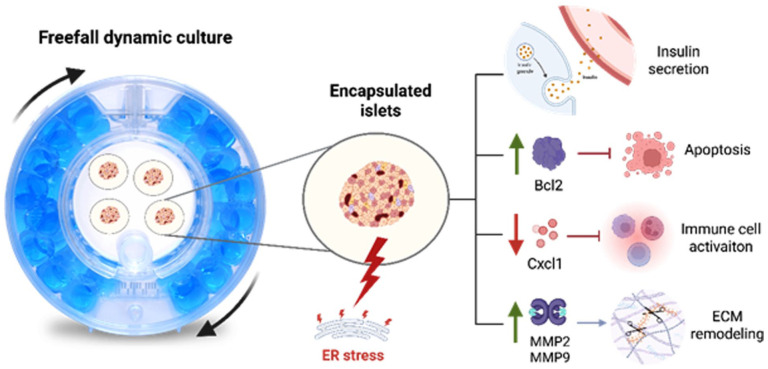
Encapsulated human pancreatic islets were cultured in a free-fall dynamic system that simulates a low-shear environment, enhancing nutrient and oxygen exchange. Upon exposure to endoplasmic reticulum (ER)-stress, dynamic culture promoted a protective and adaptive cellular response, either in alginate-only or in extracellular matrix (ECM)-containing microcapsules. Dynamic culture enhanced insulin secretion, indicating improved β-cell glucose responsiveness. It reduced the expression of inflammatory chemokines, particularly CXCL1, suggesting lower immunogenic signaling. Contributed to reduced cell death, reflecting enhanced islet viability and survival. Additionally, matrix remodeling activity was elevated, as shown by increased MMP2 and MMP9 expression, indicating an adaptive extracellular matrix turnover. Created in BioRender. Silva, I. (2025) https://BioRender.com/nf8b3yv

## Introduction

Pancreatic β-cells are highly specialized endocrine cells, which are uniquely capable of continually sensing nutrient status and responding with precise insulin secretion. Given their specialized nature and central role in maintaining glucose homeostasis, any disruption in β-cell function or survival can lead to severe metabolic disturbances.^1,2^ One prominent example is Type 1 diabetes (T1D), an autoimmune disease characterized by the selective destruction of insulin-producing pancreatic β-cells, resulting in insufficient insulin secretion and chronic hyperglycemia.^3^ Despite advancements in insulin therapy, pancreatic islet transplantation remains one of the most promising therapeutic alternatives for restoring endogenous insulin production and normalizing blood glucose levels in patients with T1D.^4^

Although pancreatic islet transplantation holds significant promise as a therapeutic strategy, isolated islets encounter multiple challenges during the isolation procedures, ex vivo maintenance, and transplantation, significantly contributing to β-cell apoptosis and functional impairment.^5,6^ Among these challenges, endoplasmic reticulum (ER) stress emerges as a critical factor determining β-cell viability, driven by conditions such as nutrient deprivation, hypoxia, and inflammatory responses encountered during the islet isolation procedures, ex vivo maintenance, and subsequent engraftment.^7,8^ The ER is responsible for protein folding and calcium homeostasis, and its dysregulation triggers the unfolded protein response (UPR).^9^ While initially protective, sustained or excessive ER-stress can shift the balance toward pro-apoptotic signaling pathways, ultimately compromising graft function and survival.^2,10^

To enhance islet engraftment and function, microencapsulation within semi-permeable microcapsules has been proposed as a strategy to protect transplanted islets from direct immune cell attack, thereby reducing or potentially eliminating the need for immunosuppressive drugs.^11^ Although encapsulation shields transplanted islets from an autoimmune attack, it does not fully address non-immune stressors encountered post-transplantation.^12^ Incorporating extracellular matrix (ECM) components into these microcapsules could further enhance islet survival, given the crucial role of ECM in supporting cellular viability and functionality within the native pancreas microenvironment.^13,14^ Indeed, enzymatic digestion during islet isolation disrupts not only vascular connections but also the supportive extracellular matrix (ECM). The loss of ECM compromises critical cell-matrix interactions that regulate cell adhesion and survival pathways, thereby negatively impacting islet viability and function.^15,16^

In parallel, pre-conditioning isolated islets under free-fall dynamic culture conditions prior to transplantation emerges as a potential strategy to enhance their resilience to the challenging post-transplantation environment.^17,18^ Dynamic free-fall culture systems provide gentle and continuous fluid motion characterized by low shear stress, promoting efficient oxygenation and nutrient exchange.^19–21^ These conditions closely mimic the physiological environment experienced by pancreatic islets in vivo and can allow an improved cellular metabolism and enhanced overall islet functionality. Furthermore, dynamic culture systems support higher-density cell cultures,^22^ enabling the maintenance of a greater number of viable and functional islets prior to transplantation.

Considering these factors, strategies that integrate ECM supplementation within microcapsules and dynamic pre-conditioning may represent a combined approach to enhance the viability, functionality, and survival of pancreatic islets post-transplantation, potentially leading to improved outcomes in clinical islet transplantation therapies for T1D.

## Materials and methods

### Primary human islet

Pancreatic islets obtained from deceased donors were processed and isolated by the Leiden University Medical Center Islet Isolation Laboratory. All related procedures involving human islets were approved and performed according to the Dutch code for proper secondary use of human tissue, as established by the Dutch Federation of Medical Scientific Societies. Table 1 provides an overview of the donor characteristics and relevant information for the islets used in this study. Before encapsulation, primary human islets were washed 3 times and maintained in CMRL 1066 medium (Pan Biotech, Germany), supplemented with 1 mM L-glutamine (Thermo Fisher Scientific, Netherlands), 50 U/mL penicillin, 50 mg/L streptomycin (Thermo Fisher Scientific, Netherlands), and 10% fetal bovine serum (FBS; Serana GmbH, Pessin, Germany). Islets were maintained in Petri dishes at a density of approximately 500–700 islets per plate in 12 mL of medium for 24 or 48 h, after which they were used for encapsulation.

**Table 1.** Human islets donor information.

Patient code	Age	BMI	Gender	Blood type	Cause of death	Estimated purity (%)
PIL-01	67	26	Male	AB−	Cardiac	93–98
PIL-02	51	20	Female	A +	Cerebral Vascular	45
PIL-03	58	26	Male	O	Non-cardiac	65
PIL-04	41	31	Female	A	Euthanasia	70
PIL-05	47	26	Male	O	Non-cardiac	65

BMI: Body Mass Index.

### Microcapsule production and islets encapsulation

Alginate microcapsules were prepared using intermediate-α-L-guluronic acid (G) alginate (42% (G)-chains, 58% β-D-mannuronic acid (M)-chains, 23% GG chains, 19% GM-chains, 38% MM-chains), obtained from ISP Alginates (Girvan, UK), to a final concentration of 3,4% in Ca²⁺-free Krebs Ringers-Hepes, KRH (133 mM NaCl, 4.69 mM KCl, 1.18 mM KH_2_PO_4_, 1.18 Mm MgSO_4_.7H_2_O, 25 mM HEPES, 2.52 CaCl_2_.2H_2_O). The production of alginate microcapsules containing ECM was carried out by mixing 70 µg/mL of MatriXpec™ Thermo porcine-derived pancreas tissue (Tissuelabs, Switzerland, cat#MXTH-PA-10) with the 3,4% alginate solution.

Human islets were encapsulated in alginate and alginate-containing ECM microcapsules using a pressure-based droplet generator by mixing 1 mL of sodium alginate with 2000 islets. The capsules were collected in a cross-linking bath containing 100 mM CaCl_2_ × 2H_2_O (10 mM HEPES and 2 mM KCl). Encapsulated islets were then washed three times in KRH buffer. Encapsulated human islets were hand-picked and separated for each culture condition and then maintained in 3 mL of CMRL 1066 medium.

### In vitro assessment of immunogenicity using THP1-XBlue™-MD2-CD14 reporter cells

To evaluate the potential immunogenicity in vitro of alginate microcapsules in the presence and in the absence of ECM, we used the THP1-XBlue™-MD2-CD14 human monocyte reporter cell line (InvivoGen, France, cat# thpx-mdcdsp). This cell line overexpresses MD2 and CD14, key components for the Toll-like receptor (TLR) signaling, which triggers the activation of nuclear factor kappa-light-chain-enhancer of activated B cells (NF-kB). When NF-kB is activated, it induces the expression of the reporter gene SEAP (secreted embryonic alkaline phosphatase), which is secreted into the culture medium. Cells were cultured in RPMI-1640 medium supplemented with 2 mM L-glutamine, 100 μg/mL normocin, 0.5% penicillin/streptomycin, 10% heat-inactivated FBS, and 25 mM HEPES. THP1 cells, at a concentration of 1 × 10^5^ cells per well in a 96-well plate, were incubated for 24 h with either KRH buffer, 10 ng/mL lipopolysaccharides (LPS from *E. coli O111:B4*, Invivogen, France, cat#tlrl-3pelps) as a positive control, or varying amounts of empty microcapsules (5, 10, or 20 control alginate capsules or alginate with ECM capsules). After incubation, 20 μL of culture supernatant was transferred to a new 96-well plate and mixed with 180 μL of QUANTI-Blue™ Solution (InvivoGen, France). The plate was incubated for 1 h at 37°C, 5% CO_2_, and SEAP activity was measured at 650 nm using a BioTek Epoch 2 microplate reader (Agilent, Netherlands). All measurements were normalized to the KRH buffer control.

### Culturing strategies

Immediately after selection by hand picking, the encapsulated human islets were equally divided into two culture conditions: static and dynamic.

#### Static culture

Encapsulated islets, approximately 100–200 islets per vessel, were maintained for 5 days in a standard 100 mm non-adherent plate (VWR, The Netherlands, cat#734-2817) containing 10 mL of CMRL 1066 medium, supplemented with 1 mM L-glutamine, 50 U/mL penicillin, 50 mg/L streptomycin, and 10% FBS, and maintained at 37°C in a humidified incubator with 5% CO₂.

#### Dynamic culture

A rotating wall vessel bioreactor, Clinostar (CelVivo, Isogen Lifescience, the Netherlands, #10004-12) was used to provide a free-fall culture condition under low physiological fluid shear. The Clinostar bioreactor vessel was calibrated prior to islets transfer according to the manufacturer’s instructions. To provide a humid environment, polymeric beads located in the outer ring of the vessel were hydrated. The culture compartment was prepared by adding 5 mL of Anti-Adherence Rinsing Solution (Stemcell Technologies, USA, cat#07010) through the top port of the bioreactor using a 10 mL syringe and sterile needle. The bioreactor was rotated inside the Clinostar incubator at 15 rotations per minute (rpm) for 5 min to coat the chamber. After coating, the anti-adherence solution was removed, and the chamber was washed twice with phosphate-buffered saline (PBS). Before cell transfer, CMRL 1066 medium, supplemented as previously described, was added to the bioreactor through the top port and rotated at 15 rpm for at least 2 h to complete the calibration. The medium was then removed, and the encapsulated islets suspension was transferred into the bioreactor by the top port and incubated in the Clinostar incubator at a rotating speed depending on the number and size of the capsules which varied from 15 to 18 rpm in order to keep them in a constant free fall. Encapsulated islets were maintained for 5 days at 37°C in a humidified incubator with 5% CO₂.

### Endoplasmic reticulum (ER) stress exposure in human islets

**Figure 1.**
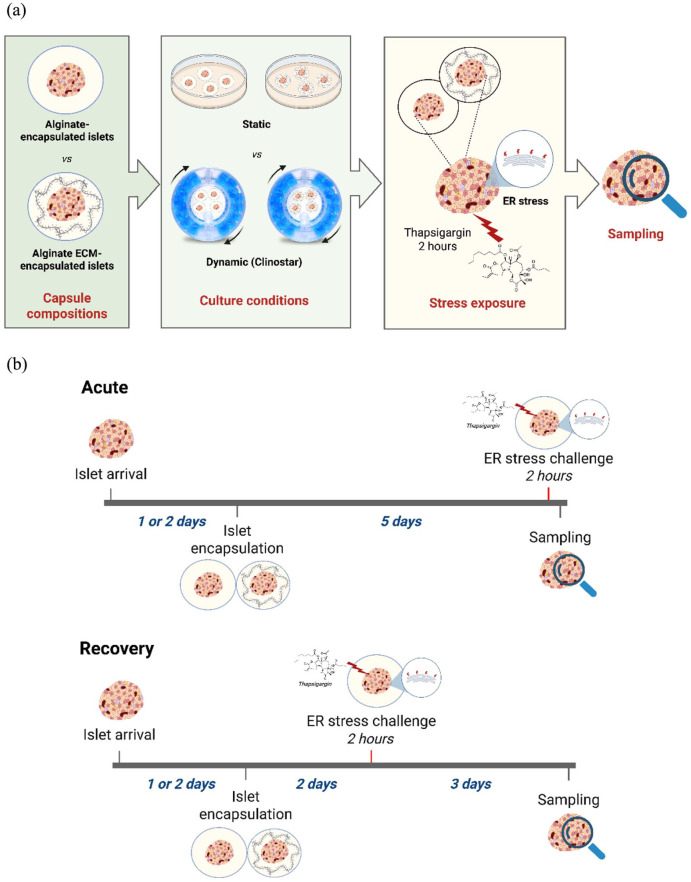
Schematic overview of human islet encapsulation, culture conditions, and stress exposure. (a) Graphical representation of the experimental workflow, including capsule compositions (alginate-only and alginate containing extracellular matrix), culture conditions (static and dynamic), and stress exposure regimen (endoplasmic reticulum, ER, stress challenge) followed by sampling for analysis. (b) Timeline and experimental design of the stress exposure regimen, divided into acute and recovery treatment conditions. In the acute treatment condition, islets were encapsulated and maintained in culture for 5 days before undergoing a 2-h of Thapsigargin challenge immediately prior to sampling. In the recovery condition, islets were encapsulated and cultured for 2 days before being exposed to 2.5 µM Thapsigargin for 2 h, followed by a recovery period until sampling on day 5. Created in BioRender. Silva, I. (2025) https://BioRender.com/nf8b3yv

The treatment scheme is illustrated in Figure 1(a). Human islets were separated into two groups and exposed to distinct stress conditions, referred to as acute and recovery (Figure 1(b)). For the acute condition, encapsulated islets in either alginate-only (Alg) or alginate containing ECM (Alg + ECM) and maintained under static or dynamic conditions were cultured in supplemented CMRL 1066 for 5 days and then treated with 2.5 µM Thapsigargin (Merck Life Science, Netherlands, cat#T9033) for 2 h to simulate acute ER-stress. Following treatment, the islets were washed three times with PBS to remove residual Thapsigargin before being collected for further experiments. For the recovery condition, islets encapsulated in either alginate-only (Alg) or alginate containing ECM (Alg + ECM) and cultured under static or dynamic conditions were treated on day 2 with 2.5 µM Thapsigargin in supplemented CMRL 1066 medium for 2 h. After treatment, the medium was removed, and the islets were washed three times with PBS to ensure the removal of residual Thapsigargin. The islets were then transferred to fresh culture vessels and returned to their original incubation conditions to recover until day 5, when they were harvested for downstream analysis.

### Cell viability assays

#### Intracellular ATP quantification

Adenosine triphosphate (ATP) levels were measured using the CellTiter-Glo^®^ 3D Cell Viability Assay (Promega, USA, cat#G9681). For each experimental replicate, five encapsulated islets were hand-picked from each experimental condition, with each islet placed in an individual well of a 96-well plate for analysis. To lyse the islets and determine total ATP content, 100 µL of CellTiter-Glo was added to each well. The plate was wrapped in aluminum foil and placed on an orbital shaker for 30 min at 800 rpm to ensure thorough lysis of the islets and homogenization of the samples. After the incubation period, 50 µL of the well content was transferred to an opaque 96-well plate suitable for luminescence measurements (Greiner Bio-one, Germany, cat#655090). Luminescence was then measured using the SynergyHT microplate reader (BioTek).

#### Live and dead viability assay

Live and dead staining was performed using the Live/Dead Viability/Cytotoxicity Kit (Thermo Fisher Scientific, Netherlands, cat#L3224). Islets were incubated with 1 mM calcein-AM and 2 mM ethidium homodimer-1 (EthD) for 30 min at 37°C in a humidified incubator with 5% CO₂. Following incubation, the staining solution was removed, and the islets were washed four times with KRH buffer to eliminate excess dye before imaging. Images were acquired using a confocal microscope (TCS SP8, Leica) equipped with an HC PL FLUOTAR 20x/0.5 dry objective. Excitation was achieved using OPSL lasers at 488 nm (for calcein-AM) and 552 nm (for EthD), with emission detected in the green channel (PMT1:494–547 nm) and red channel (PMT2: 595–647 nm), respectively.

Image analysis was conducted using the ImageJ software (National Institutes of Health, USA). Two macros were employed for processing. The first macro (NickCondon/fiji_IMB_ChannelMerge.git) merged single-channel images into a stacked image. Thresholds for the red and green channels were then established using a sample of randomly selected images. The second one, a custom-made macro, processed the stacked images and quantified the area of positive fluorescence for each channel. The proportion of dead cells was calculated as the percentage of red fluorescence area relative to the total fluorescence using the following formula:

$$\%Cell\;death=\frac{Total\;red\;area}{Sum\;of\;total\;areas\;(red+green)}\times 100$$

### Glucose-stimulated insulin secretion

Encapsulated human islets were submitted to different concentrations of glucose in order to assess their capacity to respond by secreting insulin. Twenty to twenty-five encapsulated islets from each condition were preincubated in a low glucose solution (0.25% bovine serum albumin with 2.75 mM glucose in KRH buffer) for 1.5 h in a shaking water bath at 37°C. Following this preincubation, the encapsulated islets were incubated with a low glucose solution for 1 h, followed by 1h stimulation with a high glucose solution containing 16.5 mM glucose and 110 µM 3-Isobutyl-1-methylxanthine (IBMX; Merck Life Science, Netherlands, cat#I5879). Finally, the islets were exposed to a low glucose solution again for 1 h. After each stimulation, the supernatant was collected and immediately stored at −20°C for posterior analysis. Insulin concentrations in the supernatants were determined using a human insulin Enzyme-Linked Immunosorbent Assay (ELISA) (Mercodia, Sweden, cat#10-1113-01), following the manufacturer’s instructions. To normalize the insulin secretion, the amount of insulin released was expressed relative to the DNA content of the islets. The DNA content was quantified using the Quant-iT PicoGreen dsDNA assay kit (Invitrogen, Thermo Fisher Scientific, Netherlands, cat#P7589).

### Quantitative reverse transcription polymerase chain reaction (qRT-PCR)

To assess gene expression in encapsulated human islets, quantitative reverse transcription PCR (qRT-PCR) was performed. The capsules of 100–300 encapsulated islets were dissolved using an EDTA buffer (50 mM EDTA and 10 mM HEPES) prepared in RNase-free water. Initially, the encapsulated islets were washed with cold DPBS (without Ca²⁺ and Mg²⁺) to remove residual medium. The EDTA solution was then added, and the samples were vortexed to facilitate capsule dissolution. Once the capsules were fully dissolved, the islets were centrifuged to pellet the cells. Total RNA was subsequently extracted from the isolated islets using the Qiazol kit (Qiagen, Netherlands, cat#79306), following the manufacturer’s instructions. The RNA concentration was determined using a BioTek Take3 plate (Agilent, Netherlands #TAKE3-SN), and read using the SynergyHT microplate reader (BioTek). RNA was reverse transcribed to cDNA using a 2-step cDNA synthesis protocol. First, for substrate linearization 10 mM deoxynucleoside triphosphate (dNTP; Promega, cat# U1511) and 0.5 μg/μL random primers (Promega, Netherlands cat#C1181) were added into the RNA sample and heated at 70°C for 5 min using MJ Research PTC-200 Thermal Cycler (Marshall Scientific, Netherlands cat#MJ-200). Subsequently, Moloney Murine Leukemia Virus Reverse Transcriptase set (M-MLV RT; Promega, cat#M1701) and RNasin^®^ Ribonuclease Inhibitor (Promega, Netherlands cat#N2111) were added, and samples were run at 25°C for 10 min, 37°C for 50 min and 70°C for 15 min.

Gene expressions levels were determined by qPCR (QuantStudio 7 Flex, Applied Biosystems™, Thermo Fisher Scientific, Netherlands cat #4485701) using the SYBR Green PCR Master Mix (Applied Biosystems™, Thermo Fisher Scientific, Netherlands cat# 4309155) and the gene-specific primer pairs listed in Table 2. Relative gene expression was normalized to the geometric mean of hypoxanthine-guanine phosphoribosyl transferase (HPRT) and hydroxymethylbilane synthase (HMBS), using the comparative cycle threshold method (2^ΔΔCq^).^7^

**Table 2.** List of primers used for qPCR.

Gene	Forward primer (5′–3′)	Reverse primer (5′–3′)
*HPRT*	TCATTATGCTGAGGATTTGGAAAG	GGCCTCCCATCTCCTTCATC
*HMBS*	ACGGCTCAGATAGCATACAAGAG	GTTACGAGCAGTGATGCCTACC
*CXCL1*	TGGCTTAGAACAAAGGGGCTT	GGTAGCCCTTGTTTCCCCC
*IL8*	GCTTCCTCTCGCAACAAACT	TGATGCAATTGTCTTCTACTGGTT
*HSPA5*	TCCTGCGTCGGCGTGT	GTTGCCCTGATCGTTGGC
*MMP2*	AGCTCCCGGAAAAGATTGATG	CAGGGTGCTGGCTGAGTAGAT
*MMP9*	CACGCACGACGTCTTCCA	AAGCGGTCCTGGCAGAAAT
*BAX*	CCCCGAGAGGTCTTTTTCCG	TGGTTCTGATCAGTTCCGGC
*BCL2*	GGTGAACTGGGGGAGGATTG	GTGCCGGTTCAGGTACTCAG

### Immunofluorescence staining

Encapsulated islets from each experimental group were collected for immunofluorescence staining for the ECM proteins laminin and collagen IV, and actin filaments were stained with phalloidin. The capsules were dissolved as described above and the islet cells were fixed in 4% (v/v) paraformaldehyde (Sigma-Aldrich, St. Louis, MO, USA) for 30 min at room temperature. After fixation, samples were washed twice with DPBS, 15 min each, and dehydrated sequentially in 50% and 70% ethanol, 15 min each. Islets were then embedded in paraffin and sectioned at 4 µm thickness onto glass slides. For antigen retrieval, slides stained for laminin and phalloidin were treated with 1 mg/mL pronase in PBS for 15 min at room temperature. Slides stained for collagen IV underwent heat-induced retrieval using citrate buffer at 100 °C for 15 min. All slides were washed three times in PBS. Blocking was performed for 30 min using either 5% goat serum and 1% BSA in PBS (for laminin and phalloidin) or 1% Elk and 0.5% Tween-20 in PBS (for collagen IV). Primary antibody incubations were carried out overnight at 4°C with rabbit anti-laminin (1:200, Abcam, Netherlands, cat#ab11575), phalloidin-Alexa Fluor™ 555 (1:500, Abcam, Netherlands cat#ab176756), or goat anti-collagen IV (1:100, Southern Biotech, Netherlands cat#1340-01). After three PBS washes, the samples were incubated with secondary antibodies for 45 min with donkey anti-rabbit Alexa Fluor™ 647 (1:500, Thermo Fisher, Netherlands cat#A31573) for laminin and donkey anti-goat Alexa Fluor™ Plus 647 (1:500, Thermo Fisher, Netherlands cat#A32849) for collagen IV. Nuclei were stained with DAPI (1:500 in PBS), followed by three PBS washes. The slides were mounted using Citifluor™ (Electron Microscopy Sciences, Hatfield, PA, USA) and sealed with nail polish. Images were acquired using a Leica SP8 confocal laser microscope (Leica Microsystems, Wetzlar, Germany) with a 64×/1.4 oil DIC objective. Phalloidin was excited at 555 nm (emission 565–605 nm), laminin and collagen IV at 646 nm (emission 652–700 nm), and DAPI at 405 nm (emission 410–483 nm).

### Statistical analysis

All data are presented as mean ± standard error of the mean (SEM). The Shapiro-Wilk test was used to assess data normality. Two-way ANOVA was used to assess the effects of culture condition and ECM presence. Šídák’s and Dunnett’s post-hoc multiple comparison tests were applied to compare the different conditions, as specified in the figure legends, with α = 0.05. Statistical analyses were conducted using GraphPad Prism (version 10.1.2), with *p*-values < 0.05 considered statistically significant.

## Results

### ECM incorporation into alginate capsules does not significantly increase NF-κB activation

**Figure 2.**
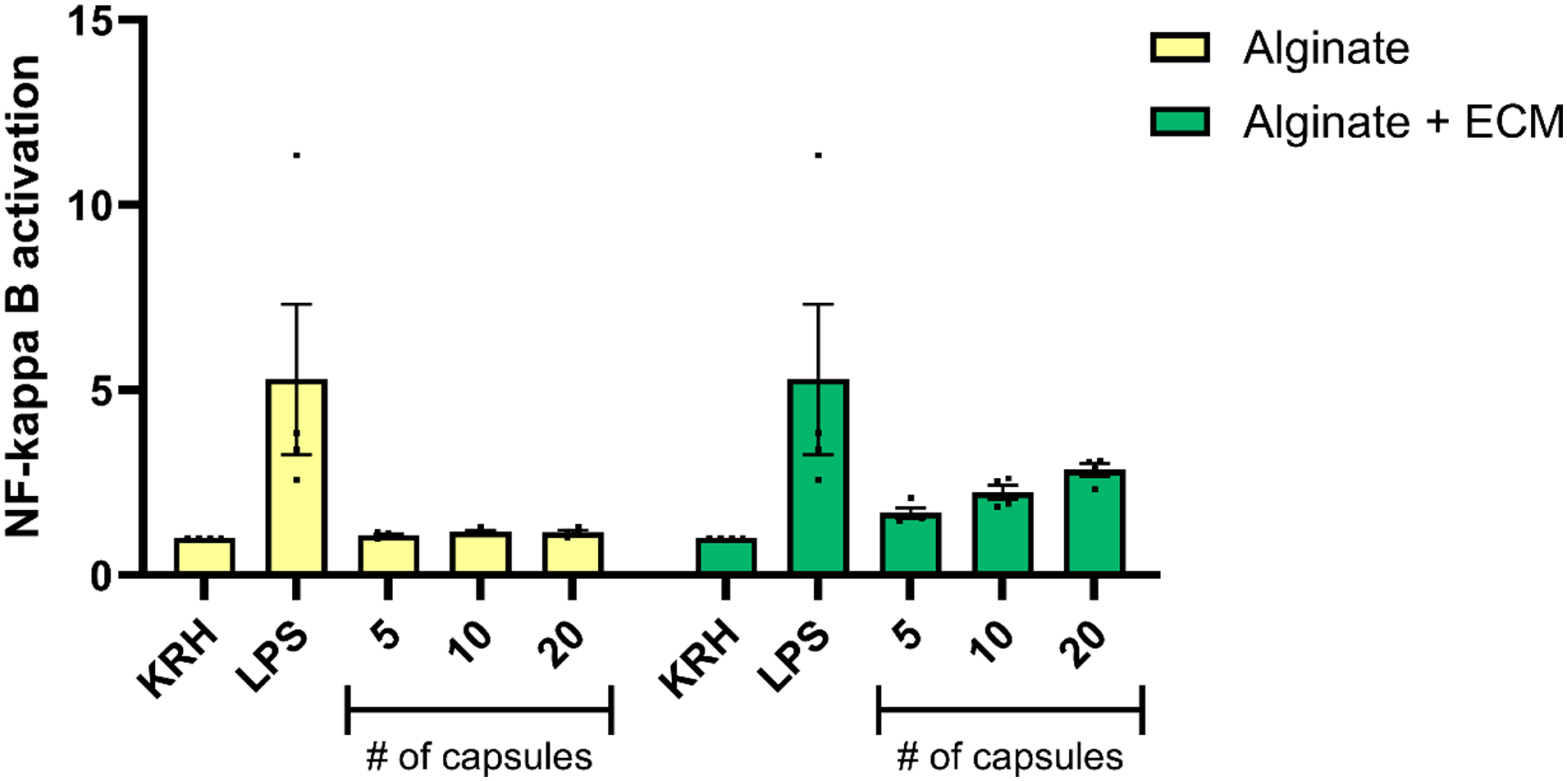
NF-κB activation in THP-1 reporter cells following incubation with empty alginate or alginate + ECM capsules. THP-1 reporter cells were incubated with 10 ng/mL lipopolysaccharides (LPS) as a positive control, or with varying numbers (5, 10, or 20) of empty microcapsules composed of either alginate-only or alginate + ECM. NF-κB activation was measured through secreted embryonic alkaline phosphatase (SEAP) activity in the culture medium after 24 h of incubation. Data are presented as mean ± SEM (*n* = 3) and normalized by the negative control, KRH buffer. Statistical analysis was performed using two-way ANOVA followed by Šídák’s multiple comparisons test.

To investigate whether the addition of decellularized porcine pancreatic ECM (dECM) to the capsule formulation could impact the immune response in vitro, we examined the activation of the NF-κB signaling pathway, an established marker of inflammatory response. This was done by incubating empty capsules with an NF-κB reporter THP-1 monocytic cell line. NF-κB activation was subsequently quantified to determine the degree of immune stimulation elicited by the modified capsule composition (Figure 2). Our results show no significant modulation of NF-κB pathway activation in response to capsules regardless of the presence of porcine pancreatic dECM. However, a dose-dependent increase trend toward increased NF-κB pathway activation was observed in capsules containing the dECM compared to alginate-only capsules.

### Free-floating dynamic culture maintains viability and suggests enhanced responsiveness of encapsulated islets in the absence of ER-stress

**Figure 3.**
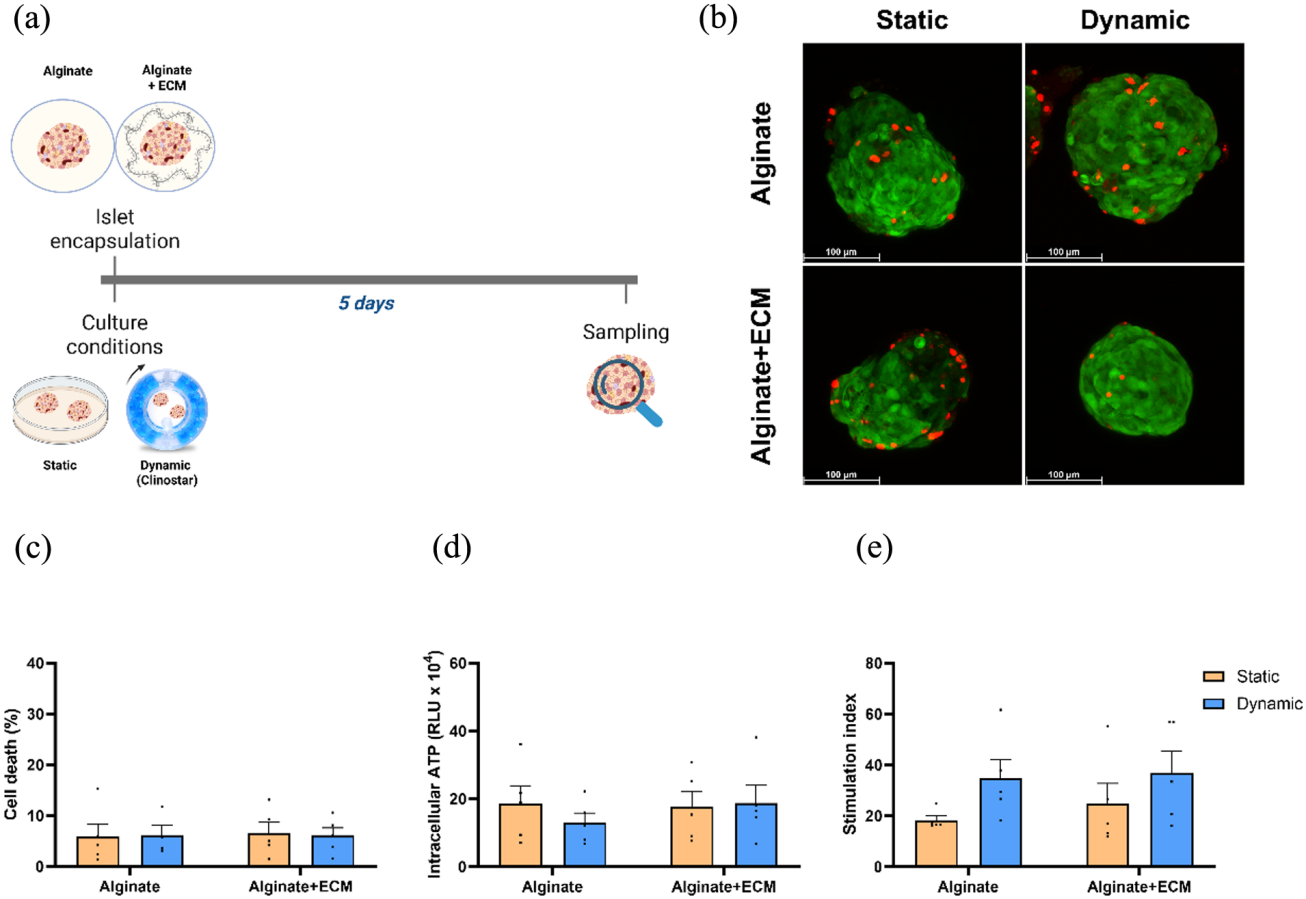
Viability and functional assessment of encapsulated human islets in alginate and alginate + ECM after 5 days of culture under static and dynamic conditions in the absence of stressors. (a) Schematic representation of the experimental design. Human islets were encapsulated in alginate or alginate + ECM and maintained in either static or dynamic culture for 5 days before assessment. Created in BioRender. Silva, I. (2025) https://BioRender.com/nf8b3yv (b) Representative images of live and dead cell staining for each capsule composition and culture condition after 5 days. Green indicates live cells stained with calcein AM, while red indicates dead cells stained with Ethidium Bromide (scale bar = 100 μm). (c) Percentage of cell death percentage measured by live and dead staining, quantified using the ImageJ software. (d) Intracellular ATP levels measured by CellTiter-Glo^®^ 3D. (e) Stimulation index calculated as the ratio of insulin secretion under high glucose versus low glucose conditions. Data are presented as mean ± SEM (*n* = 5). Two-way ANOVA followed by Šídák’s multiple comparisons test, comparing static and dynamic conditions within each capsule composition.

Free-fall dynamic culture was shown to improve cell viability and functionality by enhancing nutrient exchange and better mimicking physiological conditions.^23,24^ To assess the impact of static and free-fall dynamic culture conditions on encapsulated human pancreatic islets in the absence of external stressors, the percentage of cell death, ATP levels, and functionality were evaluated across different capsule formulations after 5 days of culture (Figure 3(a)). Live/dead staining (Figure 3(b) and (c)) showed that the percentage of islet cell death remained similar across all conditions. Intracellular ATP levels in islets encapsulated in alginate + ECM were similar between static and dynamic culture conditions, whereas ATP levels in alginate-only encapsulated islets were 0.7-fold ± 0.24 lower under dynamic culture compared to the static culture, a difference that was not statistically significant (*p* = 0.3885) (Figure 3(d)). Our results indicate that the free-fall dynamic culture does not affect islets viability and is similar to the standard static culture.

Next, we assessed islet functionality through glucose-stimulated insulin secretion (Figure 3(e)). Our results show an increased stimulation index (high glucose/low glucose response) under dynamic culture conditions for both capsule compositions. Compared to static conditions, the stimulation index increased 1.90-fold ± 0.44 (*p* = 0.0966) in alginate-only encapsulated islets and 1.49-fold ± 0.6 (*p* = 0.2424) in alginate + ECM encapsulated islets under dynamic culture conditions. Although not statistically significant, our data suggests an increased functional responsiveness of islets cultured under free-fall conditions in the absence of external stressors.

### Dynamic culture modulates inflammatory and extracellular matrix remodeling genes in encapsulated islets

**Figure 4.**
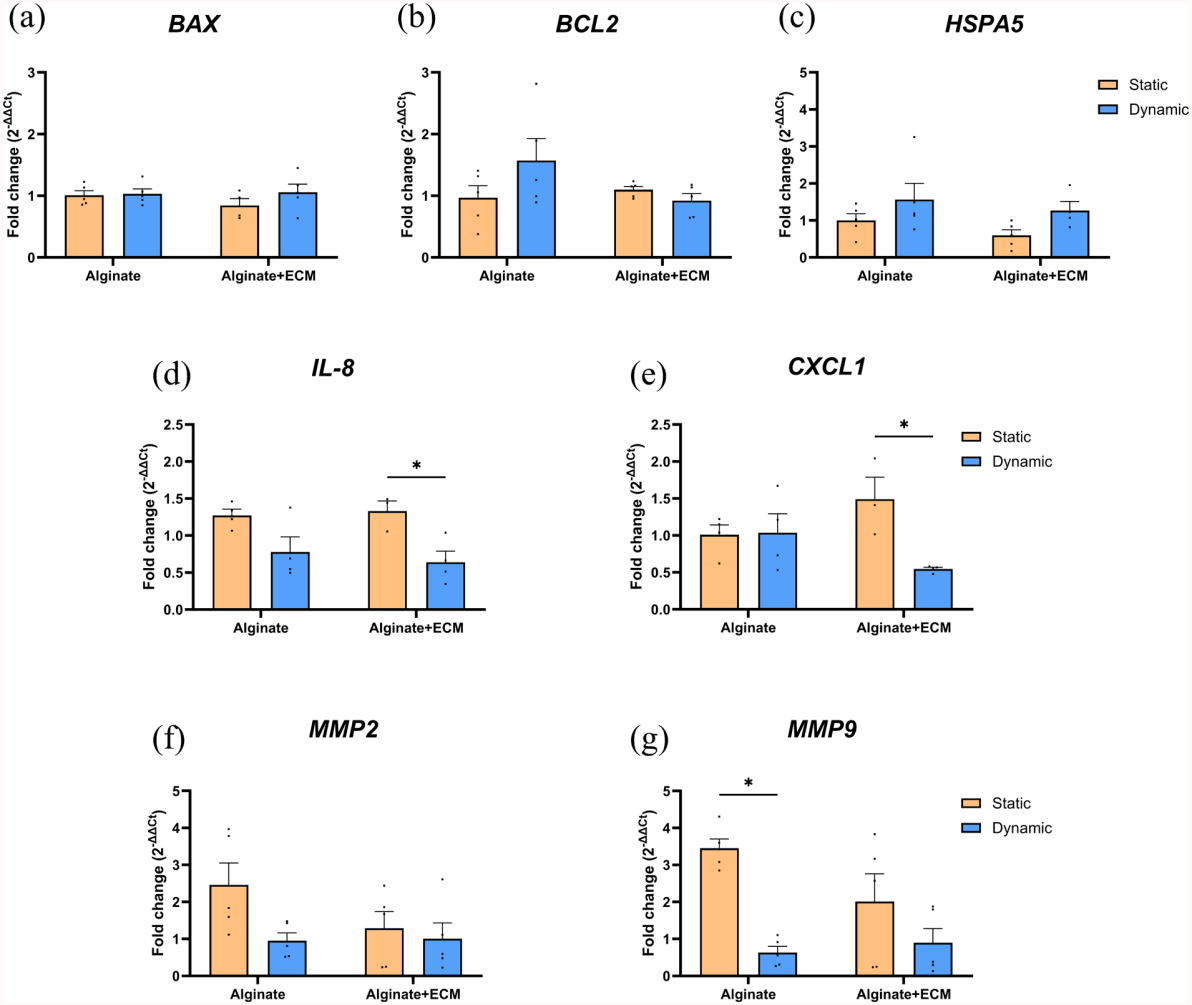
Gene expression analysis of encapsulated human islets under static and dynamic culture conditions in the absence of ER-stress. Quantification of gene expression by qRT-PCR for islets encapsulated in alginate or alginate + ECM after 5 days of culture under static and dynamic conditions. Expression levels of genes related to apoptosis and stress response (*BAX, BCL2, HSPA5*) (a–c), inflammatory response (*IL8, CXCL1*) (d and e), and extracellular matrix remodeling (*MMP2, MMP9*) (f and g) were analyzed. Gene expression was normalized to the housekeeping genes *HPRT* and *HBMS*. Data are presented as mean ± SEM (n = 3–5). Two-way ANOVA, followed by Šídák’s multiple comparisons test for static and dynamic conditions within each capsule composition (*p < 0.05).

To assess the effects of culture conditions and capsule composition on gene expression in the absence of ER-stress, we analyzed the RNA levels of genes involved in cell stress and apoptosis regulation (*BAX, BCL2, HSPA5*), inflammatory response (*IL-8, CXCL1*), and extracellular matrix remodeling (*MMP2, MMP9*) (Figure 4).

*BAX*, a pro-apoptotic gene, showed similar expression levels across static and dynamic culture conditions in both alginate and alginate + ECM encapsulated islets. In contrast, *BCL2*, an anti-apoptotic gene, was upregulated (1.62-fold ± 0.49, *p* = 0.2963) in dynamic culture for islets encapsulated in alginate-only. *HSPA5*, a molecular chaperone involved in the unfolded protein response and ER-stress regulation, exhibited higher expression under dynamic culture conditions compared to static culture in both alginate (1.56-fold ± 0.52, *p* = 0.2513) and alginate + ECM (2.13-fold ± 0.65, *p* = 0.2038) encapsulated islets.

Regarding the inflammatory response, *IL-8* expression was reduced under dynamic conditions, with a significant decrease observed in alginate + ECM encapsulated islets (0.48-fold ± 0.11, *p* = 0.0284). Similarly, *CXCL1* expression was significantly lower in alginate + ECM encapsulated islets under dynamic culture (0.37-fold ± 0.06, *p* = 0.0251). These results suggest that under free-fall dynamic conditions, the inflammatory response is attenuated in islets encapsulated in ECM-containing capsules.

For extracellular matrix remodeling genes, gene expression differences were more pronounced in alginate-only encapsulated islets between dynamic and static culture conditions. *MMP2* expression decreased by 0.39-fold ± 0.13 (*p* = 0.1313), while *MMP9* expression was significantly reduced by 0.18-fold ± 0.05 (*p* = 0.0194) in alginate-only encapsulated islets under dynamic conditions compared to static culture. These findings suggest that alginate-only encapsulated islets exhibit greater sensitivity to culture-induced extracellular matrix remodeling.

### Free-fall dynamic culture reduces the loss of functionality and cell death in encapsulated islets following acute stress

**Figure 5.**
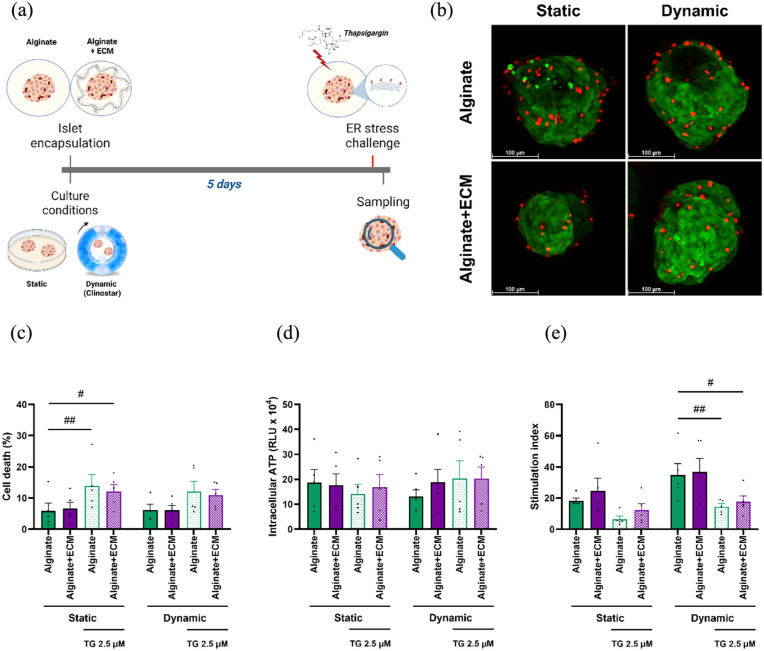
Viability and functional assessment of encapsulated human islets in alginate and alginate + ECM under static and dynamic culture conditions for 5 days followed by acute ER-stress. (a) Schematic representation of the experimental design. Human islets were encapsulated in alginate or alginate + ECM and cultured under static or dynamic conditions for 5 days before being subjected to an acute ER-stress challenge (2.5 µM Thapsigargin, (TG) for 2 h). Immediately after stress exposure, islets were analyzed for cell viability, intracellular ATP levels, and glucose-stimulated insulin secretion. Created in BioRender. Silva, I. (2025) https://BioRender.com/nf8b3yv (b) Representative images of live and dead cell staining for each capsule composition and culture condition after 5 days. Green indicates live cells stained with calcein AM, while red indicates dead cells stained with Ethidium Bromide (scale bar = 100 μm). (c) Percentage of ell death measured by live and dead staining, quantified using ImageJ software. (d) Intracellular ATP levels measured by CellTiter-Glo^®^ 3D. (e) Stimulation index calculated as the ratio of insulin secretion in high glucose versus low glucose conditions. Data are presented as mean ± SEM (*n* = 5). Two-way ANOVA followed by Šídák’s multiple comparisons test, comparing static and dynamic conditions within each capsule composition. Dunnett’s multiple comparisons test, comparing to alginate non-stressed control within culture conditions (#*p* < 0.05, ##*p* < 0.01).

To assess the ability of encapsulated human islets to withstand acute ER-stress, islets were challenged with Thapsigargin after 5 days of culture, allowing evaluation of how culture conditions and capsule compositions influence stress resistance (Figure 5(a)). Acute ER-stress increased cell death in encapsulated islets across all capsule compositions and culture conditions (Figure 5(b) and (c)), with a statistically significant change observed only in the static condition. Under static conditions, acute stress significantly increased cell death of islets in alginate-only (8.09%, *p* = 0.0031) and alginate + ECM (6.28%, *p* = 0.0219) compared to alginate-only encapsulated islets control not exposed to ER-stress. Although differences between static and dynamic conditions were not statistically significant, cell death was lower under dynamic condition in both alginate-only (1.88%) and alginate + ECM (1.15%) encapsulated islets, compared to static culture. ATP levels did not show marked variation under different culture conditions or ER-stress treatment (Figure 5(d)).

Glucose-stimulated insulin secretion was higher in dynamic culture compared to static culture when islets were challenged with ER-stressor, with the stimulation index rising by 2.23-fold ± 0.72 (*p* = 0.0669) in alginate-only encapsulated islets and 1.43-fold ± 0.56 (*p* = 0.8330) in alginate + ECM encapsulated islets, however, it did not reach statistical significance (Figure 5(e)). Under static conditions, acute stress resulted in a non-statistically significant reduction in the stimulation index for both alginate-only (0.64-fold ± 0.11, *p* = 0.5892) and alginate + ECM (0.60-fold ± 0.15, *p* = 0.4985) compared with the alginate-only control. Whereas, under dynamic culture conditions, we observed a significant lower responsiveness in alginate-only encapsulated islets by 0.42-fold ± 0.10 (*p* = 0.0033) and in alginate + ECM encapsulated islets by 0.51-fold ± 0.15 (*p* = 0.0134), compared to the alginate-only control group.

### Dynamic culture results in higher anti-apoptotic, stress-response, and matrix-remodeling gene expression in encapsulated islets under acute ER-stress

**Figure 6.**
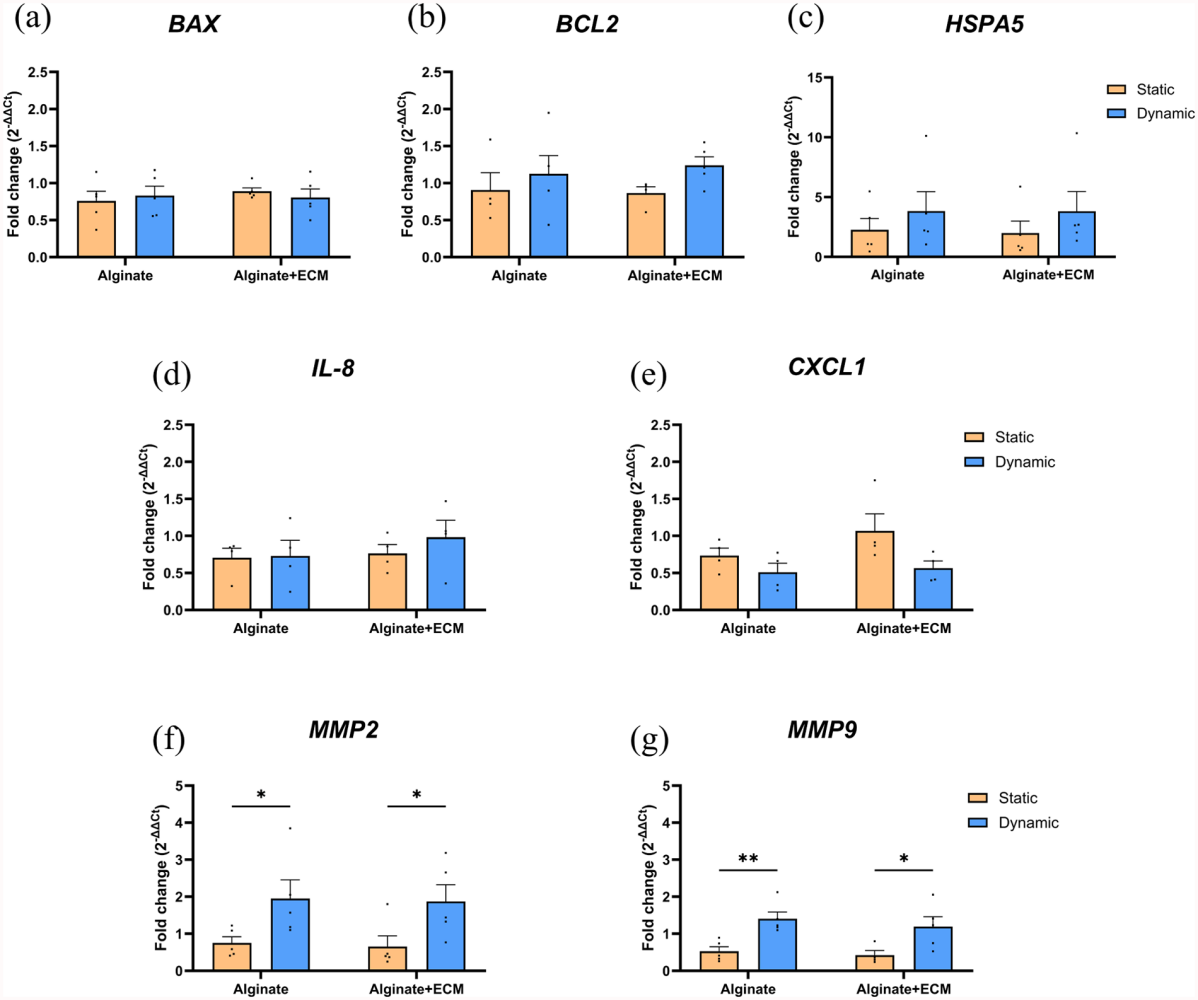
Gene expression analysis of encapsulated human islets under static and dynamic culture conditions for 5 days followed by acute ER-stress. Quantification of gene expression levels by qRT-PCR for human islets encapsulated in alginate or alginate + ECM and cultured under static or dynamic conditions for 5 days before being subjected to an acute ER-stress challenge (2.5 µM Thapsigargin for 2 h). Expression levels of genes related to apoptosis and stress response (*BAX, BCL2, HSPA5*) (a–c), inflammatory response (*IL8, CXCL1*) (d and e), and extracellular matrix remodeling (*MMP2, MMP9*) (f and g) were analyzed. Gene expression was normalized to the housekeeping genes *HPRT* and *HBMS*. Data are presented as mean ± SEM (n = 4–5). Two-way ANOVA, followed by Šídák’s multiple comparisons test for static and dynamic conditions within each capsule composition (*p < 0.05; **p < 0.01).

Gene expression analysis of encapsulated human islets after acute ER-stress (Figure 6) revealed that when compared to static condition, the dynamic culture condition resulted in higher *BCL2* expression for both capsule composition, 1.24-fold ± 0.39 (*p* = 0.6730) for alginate-only and 1.43-fold ± 0.19 (*p* = 0.3317) for alginate + ECM encapsulated islets. Additionally, dynamic culture conditions induced a higher *HSPA5* expression by 1.68-fold ± 0.99 (*p* = 0.1329) in alginate-only encapsulated islets and by 1.91-fold ± 1.26 (*p* = 0.0768) in alginate + ECM encapsulated islets compared to static conditions. However, none of these differences reached statistical significance.

Regarding the inflammatory response genes, no statistically significant difference was observed. However, the dynamic conditions induced a 1.28-fold ± 0.36 (*p* = 0.4662) increase in *IL-8* expression specifically in alginate + ECM encapsulated islets. Conversely, *CXCL1* expression was lower under dynamic conditions for both capsule compositions compared to static conditions (0.69-fold ± 0.19, *p* = 0.6005 for alginate-only and 0.53-fold ± 0.14, *p* = 0.1387 for alginate + ECM).

For extracellular matrix remodeling genes, islets exposed to acute ER-stress under dynamic culture showed a statistically significant increase in the expression of both *MMP2* and *MMP9* relative to static conditions. *MMP2* expression increased by 2.59-fold ± 0.88 (*p* = 0.0455) in alginate-only and by 2.86-fold ± 1.44 (*p* = 0.0421) in alginate + ECM encapsulated islets cultured under dynamic conditions. Similarly, *MMP9* expression showed a 2.67-fold ± 0.72 (*p* = 0.0092) increase in alginate-only and 2.83-fold ± 0.99 (*p* = 0.0293) increase in alginate + ECM encapsulated islets under dynamic culture conditions compared to static culture.

### Free-fall dynamic culture increases cell death in alginate-only islets and potentially attenuates stimulation index loss during ER-stress recovery

**Figure 7.**
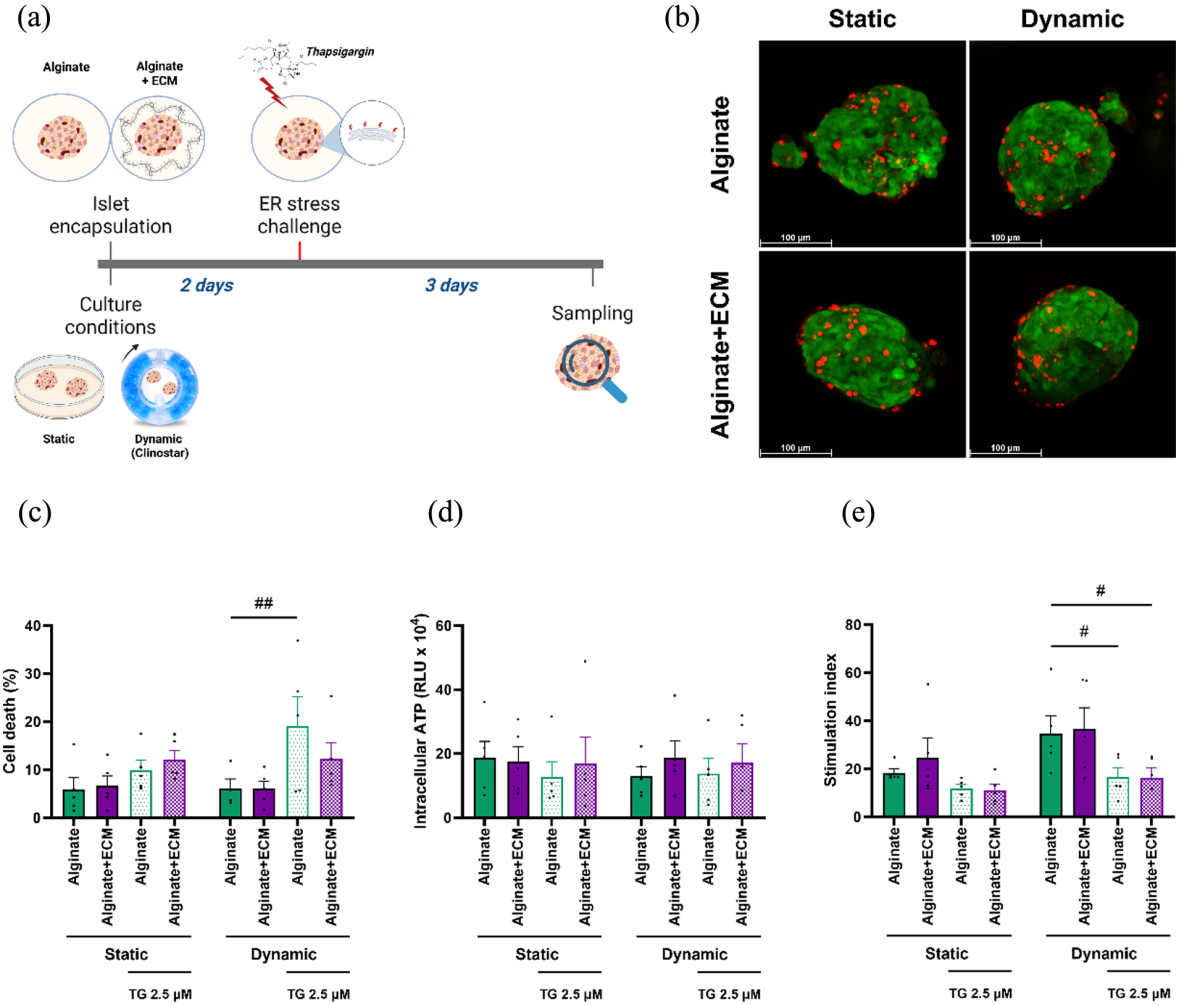
Viability and functional assessment of encapsulated human islets in alginate and alginate + ECM under static and dynamic conditions after ER-stress recovery. (a) Schematic representation of the experimental design. Human islets encapsulated in alginate or alginate + ECM were cultured under static or dynamic conditions for 2 days, exposed to ER-stress (2.5 µM Thapsigargin, 2 h), and then cultured for 3 additional days, after which cell viability, intracellular ATP levels, and glucose-stimulated insulin secretion were assessed. Created in BioRender. Silva, I. (2025) https://BioRender.com/nf8b3yv (b) Representative images of live and dead cell staining for each capsule composition and culture condition after 5 days. Green indicates live cells stained with calcein AM, while red indicates dead cells stained with Ethidium Bromide (scale bar = 100 μm). (c) Percentage of cell death measured by live and dead staining, quantified using the ImageJ software. (d) Intracellular ATP levels measured by CellTiter-Glo^®^ 3D. (e) Stimulation index calculated as the ratio of insulin secretion in high glucose versus low glucose conditions. Data are presented as mean ± SEM (*n* = 5). Two-way ANOVA followed by Šídák’s multiple comparisons test, comparing static and dynamic conditions within each capsule composition. Dunnett’s multiple comparisons test, comparing to alginate non-stressed control within culture conditions (#*p* < 0.05, ##*p* < 0.01).

To evaluate the ability of encapsulated human islets to recover from ER-stress, islets encapsulated in alginate or alginate + ECM were subjected to a 2-h ER-stress challenge after 2 days under static or dynamic culture. Following stress removal, islets remained in culture for an additional 3 days, followed by cell viability, ATP levels, and functionality assessment (Figure 7(a)).

In alginate-only encapsulated islets, ER-stress recovery led to a 9.24% increase in cell death under dynamic culture compared to static culture, although this different was not statistically significant (*p* = 0.2095). Notably, alginate-only encapsulated islets under dynamic culture exhibited significantly higher cell death when compared to the non-stressed control group (13.04%, *p* = 0.0042). Intracellular ATP levels were similar between static and dynamic culture conditions in the presence of the ER-stressor (Figure 7(d)).

The glucose-stimulated insulin secretion assay showed that the ER-stress followed by recovery affected the stimulation index under both static and dynamic cultures, with a statistically significant change observed only in the latter. In this condition, the stimulation index was higher in dynamic culture than in static culture, though the difference was not statistically significant, with values of 1.4-fold ± 0.38 (*p* = 0.2448) for alginate and 1.48-fold ± 0.51 (*p* = 0.1932) for alginate + ECM. Under free-fall dynamic conditions, islets in the ER-stress recovery condition encapsulated in either alginate or alginate + ECM showed a significantly lower stimulation index compared to the alginate control with no stress exposure, with values of 0.48 ± 0.15 (*p* = 0.0173) for alginate and 0.47 ± 0.16 (*p* = 0.0150) (Figure 7(e)).

### Subtle effects of dynamic culture and ECM incorporation on apoptosis-related and matrix remodeling gene expression during islet recovery from ER-stress

**Figure 8.**
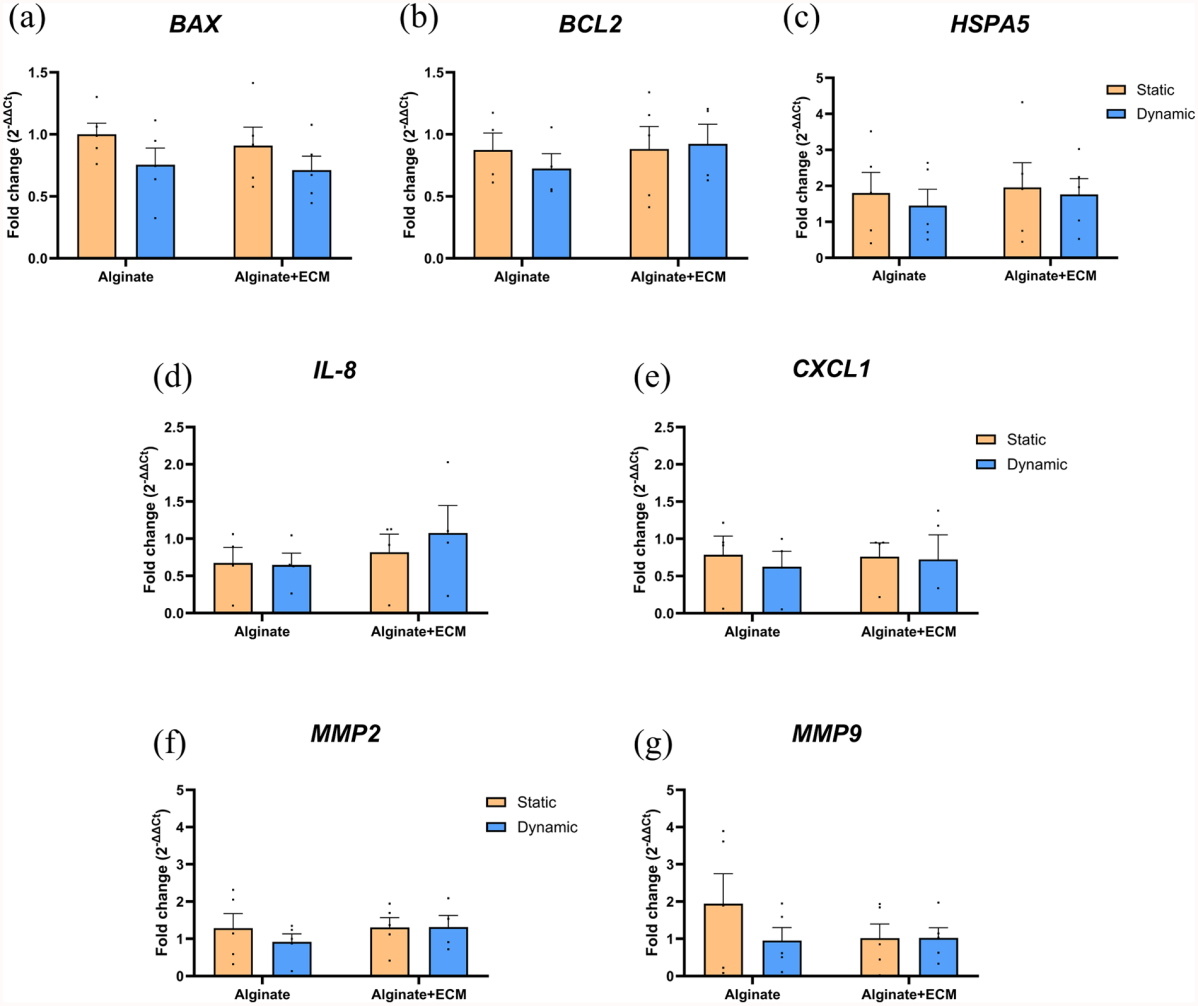
Gene expression analysis of encapsulated human islets under static and dynamic culture conditions upon ER-stress recovery. Quantification of gene expression by qRT-PCR for human islets encapsulated in alginate or alginate + ECM cultured under static or dynamic conditions for 2 days, exposed to ER-stress (2.5 µM Thapsigargin, 2 h), and then cultured for three additional days. Expression levels of genes related to apoptosis and stress response (*BAX, BCL2, HSPA5*) (a–c), inflammatory response (*IL8, CXCL1*) (d and e), and extracellular matrix remodeling (*MMP2, MMP9*) (f and g) were analyzed. Gene expression was normalized to the housekeeping genes *HPRT* and *HBMS*. Data are presented as mean ± SEM (*n* = 5). Statistical analysis was performed using two-way ANOVA, followed by Šídák’s multiple comparisons test for static and dynamic conditions within each capsule composition.

To investigate the effects of culture conditions and ECM incorporation on islet recovery after ER-stress, we analyzed the expression of selected genes in encapsulated islets maintained under static or dynamic culture (Figure 8). After 3 days of recovery, no statistically significant differences in gene expression were observed between culture conditions. However, islets under dynamic conditions showed reduced BAX expression compared to their static counterparts (0.75 ± 0.15, *p* = 0.3894 for alginate and 0.78 ± 0.18, *p* = 0.5299 for alginate + ECM). Additionally, *MMP9* expression level was higher in alginate-only encapsulated islets cultured under static conditions compared to dynamic culture (0.49-fold ± 0.27, *p* = 0.0580).

### ER-stress type differentially affects islet viability and function depending on culture conditions and capsule composition

Supplemental Figure S1 provides an overview of how acute and recovery ER-stress exposures, capsule composition, and culture conditions influence islet cell death and function. Under static culture, alginate-only encapsulated islets exhibited a significantly higher percentage of cell death following acute stress exposure, with a 2.38-fold ± 1.18 (*p* = 0.0004) increase compared to the control (Supplemental Figure 1A). In contrast, under dynamic culture conditions, cell death was more pronounced after recovery from ER-stress, showing a 3.14-fold ± 1.36 (*p* = 0.0301) increase compared to the control. For islets encapsulated in alginate + ECM, no statistically significant differences in cell death were observed across the different stress conditions or culture environments.

In terms of function, islets encapsulated in alginate + ECM showed similar stimulation index values between the ER-stress recovery and acute stress conditions in both static and dynamic cultures. In contrast, alginate-only encapsulated islets under static culture showed a greater detrimental effect on stimulation index in the acute stress condition compared to recovery (0.36-fold ± 0.11, *p* = 0.0037). Under dynamic culture, no significant difference in stimulation index was observed between acute stress and recovery for alginate-only encapsulated islets.

### Immunofluorescence staining for ECM molecules showed reduced laminin staining upon ER-stress exposure

**Figure 9.**
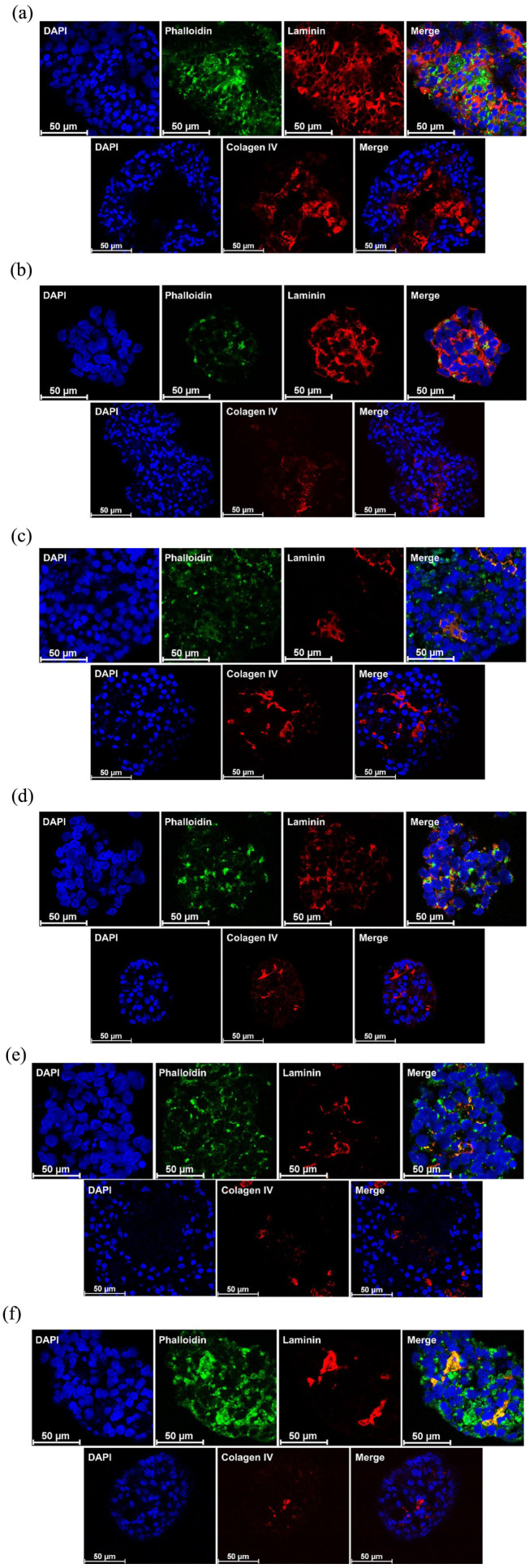
Immunofluorescence staining of alginate-encapsulated islets for laminin, collagen IV and actin filaments. (a) Representative image of alginate encapsulated islets cultured under static conditions in the absence of ER-stressor. (b) Representative image of alginate encapsulated islets cultured under dynamic conditions in the absence of ER-stressor. (c) Representative image of alginate encapsulated islets cultured under static conditions followed by acute ER-stress. (d) Representative image of alginate encapsulated islets cultured under dynamic conditions followed by acute ER-stress. (e) Representative image of alginate encapsulated islets cultured under static conditions upon ER-stress recovery. (f) Representative image of alginate encapsulated islets cultured under dynamic conditions upon ER-stress recovery. Samples were stained for cell nuclei (blue), laminin (red), phalloidin (green), and collagen IV (red) and images were captured in a confocal microscope (Leica SP8).

To gain insights into how differential MMP expression might relate to changes in islet microarchitecture, particularly among alginate-encapsulated islets under static and dynamic conditions, we performed immunofluorescence staining for key ECM components, laminin, and collagen IV, as well as phalloidin to visualize actin filament organization. Representative images are shown in Figure 9 for control (A, B), acute (C, D), and recovery (E, F) conditions. Although this analysis was limited to a single biological replicate and is qualitative in nature, some trends were visually observed. Laminin staining appeared more uniformly distributed across islets under control conditions but was reduced and more regionally confined following ER-stress exposure in both static and dynamic cultures. Under acute stress, islets under dynamic conditions (Figure 9(d)) appeared to retain a more widespread laminin distribution, when compared to their static counterparts, although under static conditions, collagen IV showed a larger stained area. Phalloidin staining under recovery conditions in dynamic culture showed a visually increased filamentous signal, which may suggest enhanced cytoskeletal reorganization. For collagen IV, staining was generally limited under most conditions, but in static control samples (Figure 9(a)), signal accumulation was observed in central regions of the islets devoid of nuclear staining, potentially indicative of a loss of structural integrity or apoptotic zones with residual collagen scaffold.

## Discussion

In this study, we hypothesized that combining ECM incorporation within microcapsules with islets pre-conditioning under free-fall dynamic culture conditions could improve islet viability and function, particularly under stress conditions that mimic the transplantation environment. To test this, we evaluated how different capsule compositions and culture conditions influenced islet responses in the absence of stressors and following acute ER-stress or recovery from this stressor.

Under control conditions, without exposure to stressors, we found that free-fall dynamic culture did not adversely affect islet viability. Moreover, islets cultured under dynamic conditions potentially improve glucose responsiveness, suggesting that the free-fall dynamic environment might support better maintenance of functional capacity. One of the aspects that renders the free-fall dynamic culture systems particularly interesting is their ability to simulate features of microgravity by providing a low-shear, low-turbulence environment that enhances cell viability.^21^ This setting promotes enhanced oxygenation and efficient nutrient and waste exchange, which can collectively contribute to improved cellular metabolism and sustained β-cell responsiveness over time.^25–27^ Studies have demonstrated that pancreatic β-cell spheroids cultured under simulated microgravity conditions using a 3D clinostat significantly improved glycemic control in diabetic mouse models, achieving better outcomes than β-cells cultured under conventional 2D conditions.^28^ Another study demonstrated that a rotational cell culture system preserved the structural integrity and glucose responsiveness of primary human islets and enhanced insulin secretion by islet-like structures, when compared to conventional static culture.^29^

Additionally, under dynamic culture conditions, we observed a potential attenuation of the inflammatory response in encapsulated islets, particularly in ECM-containing microcapsules, as indicated by the reduced expression of pro-inflammatory genes *IL-8* and *CXCL1*. These chemokines play key roles in the recruitment of neutrophils and other immune cells during the early stages of inflammation^30,31^ and have been implicated in the onset of graft inflammation and early failure in β-cell transplantation.^32,33^ Their downregulation suggests that the combination of free-fall dynamic culture and ECM incorporation within the encapsulation matrix may help create a less immunogenic environment, potentially supporting graft acceptance and long-term survival. It is well recognized that ECM can modulate immune responses and protect against immune-mediated damage in transplantation settings.^15,34^ Moreover, similar free-fall dynamic culture models have demonstrated reduced immune activation, such as lower TNF-α production^35^ and decreased immunogenicity in pancreatic islets.^36^

We also observed that static culture, particularly in alginate-only encapsulated islets, may promote enhanced sensitivity to matrix remodeling cues, as indicated by the significantly elevated expression of *MMP9*. Both MMP2 and MMP9 are crucial matrix metalloproteinases involved in ECM degradation and tissue remodeling.^37^ MMP9, in particular, plays a critical role during development, wound healing, and in pathological conditions marked by inflammation, such as arthritis, diabetes, and cancer.^38,39^ Its proteolytic activity not only remodels the ECM but also contributes to the regulation of inflammation through cleavage and activation of cytokines and chemokines.^15^

The higher *MMP9* expression, along with increased *IL-8* and *CXCL1* levels observed under static culture, may be partially attributed to the reduced efficiency of nutrient and oxygen exchange, as well as limited cell–cell and cell–matrix interactions in static conditions, which can influence gene expression related to tissue remodeling and inflammation. Compared to the dynamic culture condition, immunofluorescence staining also indicated heightened actin filaments and laminin presence in islets maintained under static cultures, which may reflect a compensatory remodeling response. Notably, *MMPs* expression was lower in islets encapsulated within ECM-containing alginate capsules, suggesting that the presence of ECM can offer crucial biochemical and biomechanical cues that help stabilize the microenvironment and diminish the need for an active-matrix remodeling.

To better simulate the complex stress landscape that islets face post-transplantation, we employed both acute and recovery ER-stress models. This dual approach allowed us to evaluate both immediate and delayed responses to stress, conditions that are highly relevant in clinical transplantation settings.^40^ We found that both acute and recovery ER-stress compromised islet viability and function across all conditions. Specifically, under acute stress, matrix remodeling markers, *MMP2* and *MMP9*, showed significantly increased expression in dynamic culture conditions across both capsule compositions. This pattern contrasts with the control conditions, in which dynamic culture tended to reduce MMPs expression. One possible explanation for the increased MMP expression observed under acute ER-stress in dynamic culture is that dynamic fluid movement may introduce subtle mechanical cues which, when combined with stress exposure, amplify cellular responses, promoting cytoskeletal rearrangement and ECM turnover, ultimately leading to elevated expression of matrix remodeling enzymes. The immunofluorescence staining revealed increased laminin and actin filament signals in dynamic culture compared to the static one despite elevated MMP levels, suggesting that the remodeling process may not be solely degradative. Instead, it may reflect a regulated adaptation mechanism, promoting ECM turnover and cytoskeletal reorganization to accommodate changes in the microenvironment.

Notably, dynamic culture under acute stress was also associated with a higher stimulation index, elevated expression of *BCL-2*, reflective of anti-apoptotic signaling,^41^ and increased expression of *HSPA5*, which encodes the protective ER chaperone BiP, indicative of early unfolded protein response (UPR) activation.^42^ Furthermore, we observed a reduction of the pro-inflammatory chemokine *CXCL1*. While these gene expression differences did not reach statistical significance, in the context of complex biological systems such trends may still be meaningful. They could reflect subtle yet coordinated cellular adaptations, with the observed upregulation of MMPs under acute stress in dynamic culture more plausibly representing an active repair or remodeling process rather than a maladaptive or damaging response.

Following ER-stress and a 3-day recovery period, islets encapsulated in alginate-only capsules and cultured under dynamic conditions exhibited the highest levels of cell death, suggesting increased vulnerability of this condition during the recovery phase. We also observed that, although the stimulation index was significantly reduced under recovery conditions for both capsule compositions in dynamic culture compared to the control, glucose responsiveness remained higher than that observed under static conditions. This partial preservation of function suggests that dynamic culture may continue to confer a degree of protection to islet functionality, even after exposure to ER-stress and recovery.

When comparing outcomes between acute and recovery stress conditions, alginate + ECM encapsulated islets displayed relatively stable responses, with minimal variation in both cell death and function. In contrast, islets encapsulated in alginate-only capsules exhibited greater sensitivity to the type of stress exposure. Under static conditions, alginate-only encapsulated islets exhibited higher levels of cell death in response to acute stress. However, when cultured dynamically, these islets showed a higher proportion of cell death during ER-stress recovery. This indicates that the culture environment modulates the vulnerability of alginate-only encapsulated islets to different phases of stress. This divergence highlights the stabilizing role of ECM components within the encapsulation matrix, potentially enhancing the islets’ ability to cope with or recover from environmental stress.

Culturing cells under free-fall conditions, or rotating wall vessel devices has been associated with supporting physiologically relevant cell–cell and cell–matrix interactions, rendering this approach particularly advantageous for three-dimensional (3D) tissue culture and engineering.^43,44^ These systems are thought to provide an optimal environment for tissue formation by enabling scaffold-free 3D cell organization, addressing key challenges in tissue engineering through the development of more physiologically relevant tissue structures.^18,45,46^ Supporting this, one study reported that mice with acute liver failure showed significantly higher survival rates when implanted with tissues cultured under these dynamic conditions compared to those receiving conventionally cultured grafts.^47^

The differences we observed in this study regarding ECM remodeling and cellular responses may be explained by the multidirectional mechanical, structural, and biochemical interactions between cells and the extracellular matrix promoted by dynamic free-fall environments.^48^ This is particularly relevant in the context of islet transplantation, as enzymatic digestion during islet isolation disrupts part of the native ECM components, impairing essential adhesive and signaling interactions. Incorporating ECM into the encapsulation matrix may help restore these cues, and when combined with dynamic culture, may enhance ECM remodeling in a controlled manner ultimately contributing to improved islet stability and function in vitro.

## Conclusion

Altogether, our findings highlight the complementary benefits of combining dynamic culture and ECM incorporation in the context of islet transplantation. Free-fall dynamic culture appears to support functional adaptation of islets by enhancing glucose responsiveness and maintaining viability, even under stress conditions. At the same time, ECM incorporation contributes to the restoration of essential cell–matrix signaling and may aid in modulating inflammatory responses post-transplantation. Additionally, the dynamic system allows for higher-density and longer-term culture, offering a practical advantage for pre-transplant conditioning and in vitro studies of β-cell function. The observed protective effects were context-dependent, varying with both the nature of the stressor and the surrounding microenvironment. These findings underscore the importance of integrated strategies in the design of biomaterial-based islet transplantation platforms aimed at improving graft survival and functional outcomes.

## Supplemental Material

sj-docx-1-tej-10.1177_20417314251383295 – Supplemental material for Differential stress responses of immunoisolated human islets embedded in pancreatic extracellular matrix under static and free-fall dynamic conditions Supplemental material, sj-docx-1-tej-10.1177_20417314251383295 for Differential stress responses of immunoisolated human islets embedded in pancreatic extracellular matrix under static and free-fall dynamic conditions by Isaura Borges-Silva, Marluce da Cunha Mantovani, Minh Danh Anh Luu, Alan Gorter, Theo Borghuis, Naschla Gasaly, Mari Cleide Sogayar, Paul deVos and Marina Trombetta-Lima in Journal of Tissue Engineering
