# Supplementary material for: Differential stress responses of immunoisolated human islets embedded in pancreatic extracellular matrix under static and free-fall dynamic conditions
Source: J Tissue Eng. 2025 Oct 27;16:20417314251383295. doi: 10.1177/20417314251383295 (PMC12575931; doi:10.1177/20417314251383295)
Supplement: sj-docx-1-tej-10.1177_20417314251383295 – Supplemental material for Differential stress responses of immunoisolated human islets embedded in pancreatic extracellular matrix under static and free-fall dynamic conditions [file sj-docx-1-tej-10.1177_20417314251383295.docx]

**Differential stress responses of immunoisolated human islets embedded in pancreatic extracellular matrix under static and free-fall dynamic conditions**

Isaura Beatriz Borges Silva ^1,2,4^*, Marluce da Cunha Mantovani ^1,4,5^*, Minh Danh Anh Luu ^1^, Alan Gorter ^1^, Theo Borghuis ^2^, Naschla Gasaly ^2^, Mari Cleide Sogayar ^3,4^, Paul de Vos ^2^, Marina Trombetta-Lima ^1^

^1^Department of Pharmaceutical Technology and Biopharmacy, Groningen Research Institute of Pharmacy, University of Groningen, Groningen, The Netherlands.

^2^Department of Pathology and Medical Biology, University of Groningen, and University Medical Center Groningen, Groningen, the Netherlands

^3^Department of Biochemistry, Chemistry Institute, University of São Paulo, Brazil

^4^Cell and Molecular Therapy NUCEL Group, School of Medicine, University of São Paulo, São Paulo, Brazil

^5^Division for Support of Training, Research and Innovation (DTAPEPI), School of Medicine, University of São Paulo, Brazil

* These authors contributed equally

**Corresponding Author**

Marina Trombetta Lima, PhD

m.trombetta.lima@rug.nl

Assistant Professor - Rosalind Franklin Fellow

Department of Pharmaceutical Technology and Biopharmacy

Groningen Research Institute of Pharmacy (GRIP) - University of Groningen (RUG)

Antonius Deusinglaan 1, 9713 AV Groningen, The Netherlands

**Supplementary material**


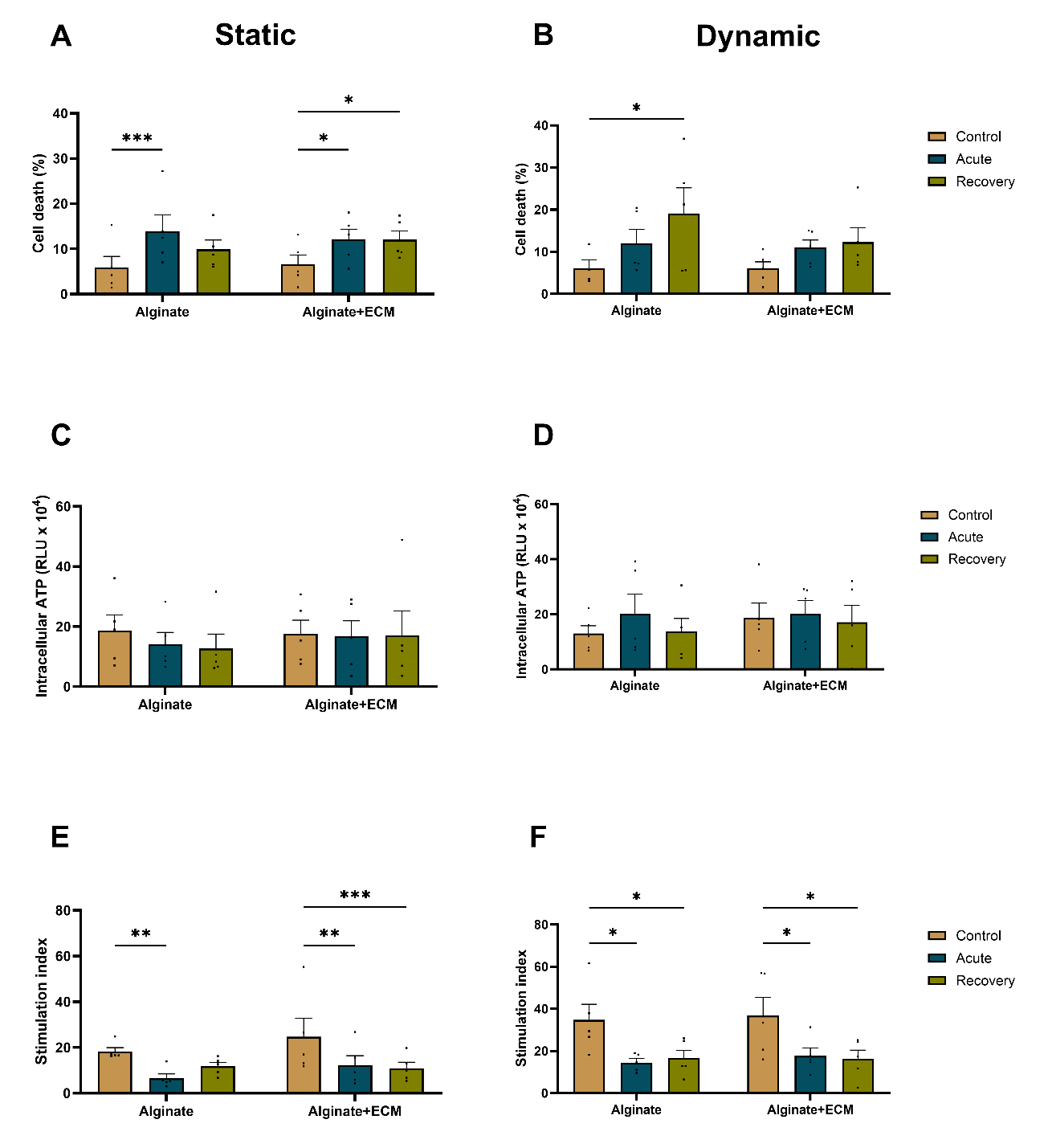


**Supplementary figure S1. Assessment of viability and functionality of encapsulated human islets under static and dynamic conditions in the absence of ER-stress (Control), following acute ER stress (Acute) and ER stress recovery (Recovery) exposure.** Human islets encapsulated in alginate or alginate+ECM were cultured under static (left panels) or dynamic (right panels) conditions and exposed to either recovery (ER stress followed by 3 days of culture) or acute (ER stress immediately prior to analysis) conditions. Control represents islets cultured without exposure to ER stress. (A, B) Cell death percentage, quantified from live/dead staining using ImageJ software. (C, D) Intracellular ATP levels, measured using the CellTiter-Glo® 3D assay. (E, F) Stimulation index, calculated as the ratio of insulin secretion in high glucose compared to low glucose conditions, assessing islet functionality. Data are presented as mean ± SEM (n = 5). Statistical analysis was performed using two-way ANOVA, followed by Tukey's multiple comparisons test (∗p < 0.05; **p < 0.01; ***p < 0.001).

**
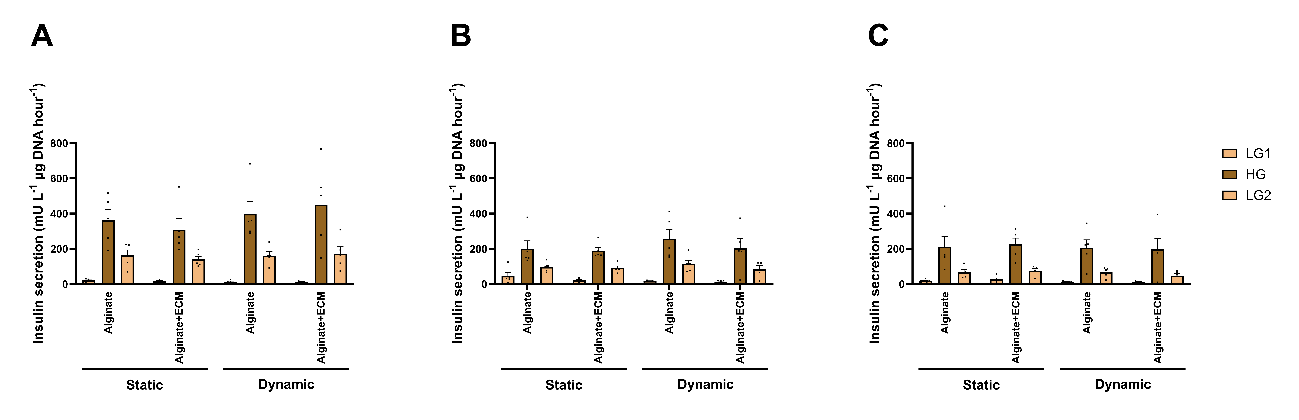
**

**Supplementary Figure S2. Glucose-stimulated insulin secretion (GSIS) of encapsulated human islets under static and dynamic culture conditions in the absence of ER stress (A), after acute ER stress (B), and following ER-stress recovery (C).** Human islets were encapsulated in either alginate or alginate+ECM and cultured under static or dynamic conditions. GSIS was assessed using a sequential glucose challenge, in which islets were first incubated in low-glucose buffer (2.75 mM; basal phase), followed by high-glucose buffer (16.5 mM; stimulatory phase) to trigger insulin release, and then returned to low-glucose buffer to assess recovery and residual secretion. Insulin secretion values were normalized to DNA content measured from the same samples after GSIS completion, using the Quant-iT™ PicoGreen® dsDNA Assay Kit (Invitrogen, Thermo Fisher Scientific). Data are presented as mean ± SEM.
